# Notes and News

**Published:** 1891-09-19

**Authors:** 


### Notes and News.

Collected and Communicated.

Early in October there will be commenced with the new volume of The Hospital a series of articles on "Medical History from the Earliest Times," and also a series on "Talks about Food." No pains have been spared to make these articles interesting, instructive, and useful to all classes of readers.

The report of the Mater Misericordiæ Hospital for the thirtieth session, 1891-92, shows progress. It is the largest general hospital in the city and has now 311 beds available for patients, and is provided with the best modern scientific appliances in every department. During the year a total of 3,613 intern patients were admitted, while no less than 26,530 extern patients were treated. A training school for nurses has been established in connection with the hospital, and private wards have also been opened. A glance at the list of past students shows how widely extended its influence has been and is as a teaching centre. The Mater Misericordiæ is wholly supported by voluntary contributions, and it is a sterling and self-evident tribute to the earnestness and ability of its working staff that it should have attained its universal repute and confidence under circumstances of the kind. It may be noted that the Bacteriological Laboratory founded in 1890 by Archbishop Walsh, who paid the expenses of its foundation, is the first that has been instituted by an Irish hospital.

The Cottage Hospital at Boscombe has begun to show tangible signs of a much needed activity. Last week the corner-stone of a new wing about to be added to this valued, but all too inadequately small, establishment was laid by Mrs. Maberly, of Christchurch. The addition, which is being built at a cost of £350, is to consist of an out-patients' room and an operating theatre combined, a waiting-room, a dispensary, and bath-room and lavatory, all absolute necessities to a well-conducted hospital however small. Other improvements equally desirable, especially two rooms for the Matron, are spoken of, if the necessary funds (£150) can be procured. It is just possible that there are many scattered over the Kingdom who owe a deep debt of gratitude to the healing breezes of Boscombe, the most bracing suburb of Bournemouth, either on their own account or that of someone dear to them. Let them gracefully requite that debt by each putting a brick at least—no such great demand on their pockets—to an edifice which is of most vital importance to their poorer brethren.

Professor Tyndall has contributed an important article to the *Fortnightly Review* on "The Origin, Propagation, and Prevention of Phthisis." For the last twenty years Professor Tyndall has been conducting experiments respecting the germ of this disease. Nine years ago he was the first to introduce to the English public those results of Professor Koch's enquiries which first made him famous. Dr. Tyndall's present paper is mostly devoted to the researches of Dr. Georg Cornet, also of the Imperial Sanitary Institute of Berlin, who has demonstrated by experiment that it is the dust in the sick-room of the consumptive patient that is pre-eminently dangerous. The Professor illustrates and enforces this truth by some facts derived from his Alpine experiences. In closing this paper he calls on the British people "not to be frightened by a number of loud-tongued sentimentalists" who oppose experiments on living animals, and who might, he says, be fairly described as "a crew of well-meaning homicides." In this matter he does not believe, with his friend Carlyle, that the English people are mostly fools, if only scientific men will now courageously make clear to the public the beneficence of their aims.

Dr. Dingwall, on the occasion of his leaving Banff to commence the practice of his profession in Fraserburgh, was presented by the Matron and nurses of Chalmers Hospital with a handsome case of surgical instruments in appreciation of his services as Lecturer to the nurses during the years 1890-91.

The thirty-fourth report of the Inspector of Reformatory and Industrial Schools of Great Britain states that the total number of schools under inspection is now 225, namely, 55 reformatory schools, 141 industrial schools, 10 truant schools, and 19 day industrial schools. The total number of juveniles under sentence of detention in these schools at the close of 1890 was 28,588, namely, 23,509 boys, and 5,080 girls. This shows an increase of 504 boys, and an increase of eight girls, as compared with the previous year. The average number of children attending day industrial schools in 1890 was 3,698. They provide education and food for children of the very poorest class, at a very low cost to the public. The schools are opened early in the morning for such children as like to go; but the regular work of the school does not begin till eight o'clock, when breakfast is provided. The children have a substantial dinner in the middle of the day, and tea before they are dismissed at six o'clock. Every attention is paid to cleanliness, and baths are supplied in every school, and are used regularly and frequently. The education is invariably well attended to, and what is attempted is very thoroughly done.

A patient of the Mary Wardell Convalescent Home for Scarlet Fever draws attention to the excellent work which is being done by it. "Although the Metropolitan Asylums Board possesses a large convalescent home at Winchmore Hill, its use is restricted to patients coming from the Board's acute hospitals. At the present time, therefore, this place is the only refuge for those who have passed through the acute stages of the disease in their own homes. The founders originally intended this institution for patients from the poorer parts of London who were unable to pay the whole cost of their maintenance, but it was subsequently decided to provide, in addition, accommodation for those who were in a position to defray their own expenses. It would be difficult to overrate the advantages both to the private and public health which an institution of this kind affords, and it is because I find it not so widely known as it deserves to be that I am troubling you with this communication." The home is situated on Brockley Hill, Stanmore, Middlesex, which is within easy driving distance of London. The water-supply is good, and the drainage, ventilation, and disinfecting apparatus are all of modern type.

It is just a year since at the quarterly court of the London Hospital, a small faction of malcontents commenced a vigorous and undeserved course of censure on the management of the hospital. The time was ill-chosen; Mr. Ind and Mr. Buxton, Chairman and Treasurer, were both young and enthusiastic, and withal men of business and common-sense, and they were backed by Sir Edmund Currie, whose downright language is calculated to make the unexact and exaggerators tremble with shame. For some time the battle waxed fierce, and it was feared some injury might be done to the funds of the hospital by all the mud-slinging that went on; but each charge was met as soon as made, and the revulsion of public feeling brought unexpected sympathy and aid to the institution. At last week's Court of Governors the tide had ebbed, the attendance was small, the proceedings unmarked by any wrangling. Dr. B. Fenwick made a few feeble moans, but Mr. Yatman sat and listened to him in silence; Mr. and Mrs. Hunter were not present. So dies away the biggest storm our voluntary hospitals have had to stand for many a long day.

